# Psychological Flow Training: Feasibility and Preliminary Efficacy of an Educational Intervention on Flow

**DOI:** 10.1007/s41042-023-00098-2

**Published:** 2023-05-23

**Authors:** Cameron Norsworthy, James A. Dimmock, Joanna Nicholas, Amanda Krause, Ben Jackson

**Affiliations:** 1 School of Human Sciences (Exercise and Sport Science), University of Western Australia, Perth, Australia; 2 Department of Psychology, College of Healthcare Sciences, James Cook University, Townsville, Australia; 3 Western Australian Academy of Performing Arts, Edith Cowan University, Perth, Australia; 4 Telethon Kids Institute, Perth, Western Australia Australia

**Keywords:** Performance, Optimal functioning, Mental skills, Flow state, Stress, Anxiety, Motivation

## Abstract

Despite there being an increasing number of applied flow studies across scientific disciplines, there exists no consistent or broadly applicable intervention to promote flow experiences. This study provides a detailed account of a new educational flow training program developed following recent advancements in the flow literature that have provided a more parsimonious understanding of flow experiences and antecedents. Guided by CONSORT guidelines for feasibility trials, we conducted a single-group, non-randomized feasibility trial of an educational flow training program (*N =* 26). We assessed participant retention, perceptions about and experiences of the program, perceptions about the flow education training, and preliminary assessments of flow as an outcome. Results broadly supported program feasibility, and participants reported positive experiences in, and perceptions of, program components. In terms of preliminary efficacy, we observed evidence of noteworthy change pre-to-post-program in flow (*d* = 0.84), performance (*d* = 0.81), competence (*d* = 0.96), well-being (*d* = 0.68), intrinsic motivation (*d* = 0.47), interest (*d* = 0.72), choice (*d* = 0.38), stress (*d* = -1.08), ability to handle stress (*d* = 0.74), and anxiety (*d* = − 0.86). These results provide preliminary evidence that it may be possible to ‘train’ flow in line with recent perspectives on a core three-dimensional flow experience (and antecedents). The study has developed a research foundation for flow intervention “curriculum” and quality standards, and for measuring results. It offers a foundation for the implementation of a larger-scale program.

**Supplementary Information:**

The online version contains supplementary material available at 10.1007/s41042-023-00098-2.

## Introduction

Following Csikszentmihalyi’s (1975) seminal work on flow, the flow state has emerged as a dominant theme in positive psychology (Seligman, 2012), producing a wealth of literature showing that flow is an optimal psychological state (e.g., Peifer & Engeser, 2021) characterised by an absorption and effortlessness in one’s subjective experience (Csikszentmihalyi, 2014). Flow is described as a valued, memorable, and highly positive experience positioned at an intersection between peak performance and peak experience (Jackson & Csikszentmihalyi, 1999; Csikszentmihalyi, 2014), and has been repeatedly linked with elevated performance (e.g., Flett 2015, Jackson & Roberts, 1992) and well-being (Haworth, 1993). As a result of propositions that flow can be controllable (72%) and restorable (81%; i.e., re-established once lost; Swann et al., 2012)—there appears to be significant interest among researchers and applied practitioners with respect to methods for promoting or ‘training individuals to achieve flow’ (also see Goddard et al., 2021).

Flow has been most frequently conceptualised using Csikszentmihalyi’s nine-dimensional model (see Jackson & Csikszentmihalyi, 1999), which outlines nine common descriptors of the flow experience—three of which (challenge-skill balance, clear goals, unambiguous feedback) were later conceptualized by Csikszentmihalyi as better reflecting preconditions (Nakamura & Csikszentmihalyi, 2009). More recently, in an attempt to overcome ongoing debate regarding construct validity, conflation of antecedents and experiential dimensions, and relational ambiguity within the nine-dimensional model (e.g., Boyd et al., 2018, Engeser & Schiepe-Tiska, 2012; Heutte et al., 2016; Norsworthy et al., 2023), Norsworthy et al. (2021) conducted a scoping review encompassing over 230 flow-related works spanning psychology, physiology, and neuroscience (and other disciplines). Based on the findings of review sources, Norsworthy and colleagues presented a parsimonious, domain-general model of flow, which separated ‘antecedents’ (i.e., a temporary state that precedes and influences the onset; Crozier et al., 2013; the term ‘antecedent’ was preferred over ‘pre-condition’ as the past literature shows these constructs as influential to the onset of flow but not necessarily as a preceding requirement in its own right, for example, the optimal level of challenge antecedent may not be relevant for non-achievement situations that may require a high level of motivation to draw one’s attention instead) from ‘experiential dimensions’, utilised language that can be utilised across scientific disciplines (enabling cross-comparative research), and identified a common over-arching core experience of flow. In line with requests for a more parsimonious theory of flow, such the proposition to identify a core flow experience containing fewer dimensions within the decennial *Advances of Flow Research* publication (Peifer et al., 2022), Norsworthy and colleagues proposed framework from the review highlighted two common antecedents (i.e., optimal challenge, high motivation) and three core experiential dimensions to flow (i.e., absorption, effort-less control, intrinsic reward). *Optimal challenge* was defined as ‘a perceived capability to meet the challenging demands of the situation’. *High motivation* was defined as ‘a high motivational force’. *Absorption* was defined as ‘a state of absorption in the task characterised by focused, undistracted attention, and a merging of action and awareness. *Effort-less control* was defined as ‘a high sense of control in which the task feels less effortful than is typical for that person, characterised by fluidity of performance and an absence of concern over losing control’. *Intrinsic reward* was defined as ‘an intrinsically rewarding experience characterised by positive valence and optimal levels of arousal’. In Norsworthy et al.’s review, the authors also observed a trend for researchers to utilise over 140 markers and measurement instruments to measure flow that, in sum, contributed to difficulties when attempting to synthesise findings and approaches. Grounded in these findings, Norsworthy et al. (2023) recently provided preliminary evidence for the theoretical and operational suitability of a domain-general Psychological Flow Scale (PFS) that measures a global latent flow factor and sub-dimensions of ‘absorption’, ‘effort-less control’, and ‘intrinsic reward’.

With regards to intervention-based flow research, Norsworthy et al. (2021) reported that applied flow studies were most prominent in psychological, neuroscientific, psychophysiological and computing disciplines, with the most frequently used samples being students, athletes, gamers, and the general population. In terms of intervention context, Norsworthy and colleagues reported that manipulating challenge level during a *computer game* scenario was the most widely deployed intervention, most likely due to the control afforded and the capacity to include physiological measures during performance. This was followed by manipulating flow antecedents within *competitive sport matches, mathematic activities*, and *educational tasks*. When not manipulating the context to be more or less challenging or motivating, psychological interventions (primarily in the context of sport) targeting arousal regulation, goal setting, self-talk, and visualisation (e.g., Nicholls et al., 2005; Pain et al., 2011; Pates et al., 2012; Koehn et al., 2014) have yielded mixed results. Other interventions using hypnosis among cyclists (Lindsay et al., 2005) and golfers (Pates & Cowan, 2013), imagery combined with music in soccer players (Pain et al., 2011), a flow education program for elite climbers (Norsworthy et al., 2017a, b), and mindfulness among athletes (Aherne et al., 2011), have demonstrated ‘positive’ results (i.e., the capacity to improve flow experiences among participants). Importantly, the vast majority of these studies have been resource intensive (e.g., requiring one-on-one in-person training) and/or specifically designed to meet the demands of the specific focal domain, making them difficult to scale or transfer across domains. In addition, and more importantly, none of the above-mentioned studies have benefited from being informed by the most recent developments in our understanding of what might be considered the most common contributors to, and elements of, the flow experience (see Norsworthy et al., 2023). In summary, therefore, although several intervention studies have demonstrated the capacity to ‘train’ flow experiences, there exists no consistent or widely-used intervention for this purpose that is informed by recent developments in the flow literature. The overarching purpose of this study therefore was to develop and evaluate the feasibility of an educational flow training program to develop a solid research foundation for intervention “curriculum” and quality standards, and for measuring results and associated outcomes.

A consistent recommendation for applied flow research is to separate flow (and the training thereof) from other similar states or outcomes such as clutch states, peak performance, play, or peak experiences (Harmison & Casto, 2012; Norsworthy et al., 2017b; Swann et al., 2017). Scholars have noted that flow is a commonly misunderstood concept (Hytonen-Ng, 2016). Whilst flow is often positively correlated with high performance (e.g., Flett, 2015)—possibly due to shared descriptors such as high concentration, clear goals, unambiguous feedback, and a high sense of control (Jackson & Csikszentmihalyi, 1999)—‘high performance’ strategies do not always promote all or any of the experiential descriptors of flow, such as a loss of self-consciousness, effort-less control, intrinsic reward, or absorption (merging of action and awareness); see Norsworthy et al., 2017b; or Swann et al.’s, 2017, delineation between clutch and flow states, for example. Therefore, although aspects of flow training may overlap with peak performance training, a ‘sensitive’ approach to manipulating flow would likely not be identical (in terms of content) to peak performance interventions. In that vein, Lindsay et al. (2005) recommended the inclusion of flow-related education (or cognitive restructuring) in flow interventions to ensure that the construct or experience (and ensuing training) is understood and able to be targeted appropriately. Similarly, Norsworthy et al. (2017a, b) recommended that education-based flow interventions should be pursued due to the positive results reported from the educational component of their flow training program, and the practical benefits of such approaches such as the suitability for widespread dissemination (see also recommendations by Swann et al., 2012). Establishing effective principles for a flow intervention is practically appealing for many professionals and practitioners. It is an important pursuit to ensure applied flow research includes more experimental research so that flow science can grow in prominence and sit alongside other more mainstream concepts, such as intrinsic motivation for example.

### The Present Study

The broad aim of this study was to develop and evaluate the feasibility of an educational flow training program that specifically targeted the two antecedents (optimal challenge, high motivation) and three experiential dimensions (absorption, effort-less control, intrinsic reward) of flow documented by Norsworthy et al. (2021). Feasibility trials are designed to provide insight into the content, delivery, and optimisation of interventions or services, and are considered a necessary first stage in program development (Eldridge et al., 2016). Feasibility studies typically include the assessment of participant retention, perceptions about the training components and program as a whole, experiences of the program, and perceptions of program effectiveness (Arain et al., 2010), and provide researchers with insight that supports the implementation of a larger-scale experimental program to examine causality. In addition to understanding these fidelity and delivery issues, we also aimed to provide insight into the preliminary efficacy or likely effectiveness in future powered trials of the newly developed flow training program. The aims of this study, therefore, were to (a) establish evidence for (or against) the feasibility of this flow training program, and (b) obtain *preliminary* insight into the potential outcomes of the training. Accordingly, we assessed participant retention, training delivery, intervention component effectiveness, participant feedback, and outcomes associated with the intervention. For the sake of preliminary efficacy assessment, outcomes included flow scores, self-rated performance, well-being, stress, anxiety, intrinsic motivation, perceived competence, interest, choice/autonomy, challenge approach, curiosity, desire to learn, enjoyment, ability to self-generate the antecedents to flow, and the ability to self-generate the experience of flow.

## Methods

### Transparency and Openness

This study is reported in line with Consolidated Standards of Reporting Trials (CONSORT) guidelines extension for randomized pilot and feasibility trials (Eldridge et al., 2016) and Journal Article Reporting Standards (JARS; see Appelbaum et al., 2018). We describe our sampling plan, all data exclusions (if any), all manipulations, and all measures in the study, adhering to the Transparency and Openness Promotion (TOP) Guidelines (Nosek et al., 2015). All data, analysis code, and research materials can be found in the article, S1, S2 (Supplementary Materials), and the Appendix 1. Data analysis is detailed below. The study protocol was approved (RA/4/20/6092) by the lead author’s institutional ethics committee prior to data collection.

### Procedure

A single intervention group trial was adopted, which is standard practice for non-randomized feasibility studies (Eldridge et al., 2016). Participants enrolled in the program through email communication with the lead researcher. Participants were asked to pick a single activity (i.e., sport) to perform and practice their training on throughout the study, and to refer to when responding to assessments. A three-hour workshop on flow was delivered separately to two groups—one remotely (*n = 5*; via Zoom) and another in-person (*n = 29*; on campus). In both settings, the first author presented the training program (see ‘program details’ below) and the workshop was structured using PowerPoint slides. In-person training followed COVID-19 safety procedures based on local government recommendations and Safe Work Australia’s Covid-19 resource kit. Following the workshop (i.e., two- and four-days after the workshop), two 1-hour follow-up question-and-answer sessions were conducted over zoom—a recording of the webinar was sent to participants (25% on average) unable to attend. Participants completed measures pre-intervention (i.e., in the days leading up to the intervention), post-workshop (i.e., within two days of the 3-hour workshop) and post-intervention (i.e., 10 days after the initial workshop, which was also 4 days after the two 1-hour follow up sessions) to assess their expectations, activity experience, intervention adherence, perceptions about the intervention, fidelity of training (e.g., participant perception on professionalism of training), and outcomes (Boot et al., 2013). An additional post-workshop survey was completed within 24 hours of the initial 3-hour workshop to specifically assess workshop-related perceptions. All measures were collected online via Qualtrics and each survey took up to 15 minutes to complete. Once exported, all data were kept securely on the first author’s personal computer. Participants were sent one group email and one personal reminder email to complete each survey.

### Recruitment

**Fig. 1:**
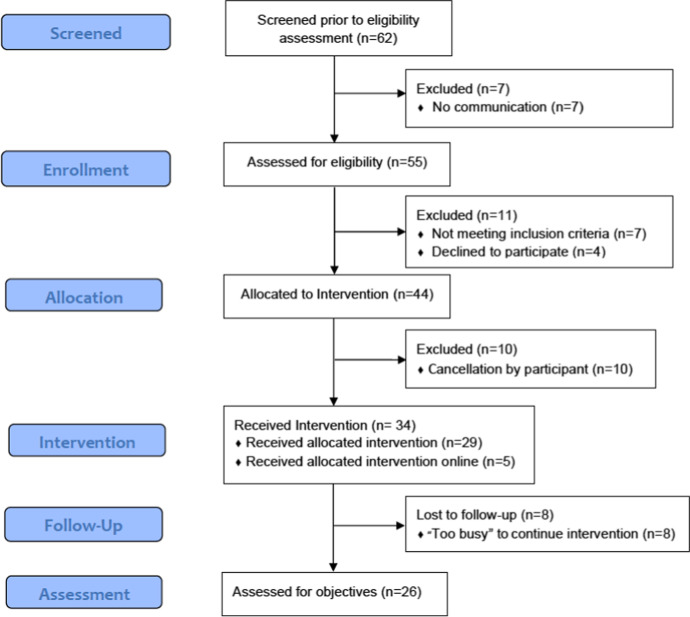
Participant flow diagram

The CONSORT flow diagram (see Fig. 1) details enrolment and intervention allocation rates. In summary, a total of 62 participants registered initial interest in the program. Overall, there was a program enrolment rate (i.e., initial interest to study allocation) of 71%, a completion rate (i.e., study allocation to study completion) of 59%, and a conversion rate (i.e., initial interest in the program to study completion) of 42%. The majority of participants who completed the initial free training but did not complete the measurement surveys indicated that they were “too busy” to fill out the forms.

### Participants

This feasibility study included 26 participants from Perth, Western Australia; 44 participants were initially allocated to the intervention but due to COVID19 constraints. Ten participants could not make the training dates, and 8 failed to fill in the data forms post-workshop due to personal situations. Twenty-six participants completed all the required measures to complete the study (see Appendix, Fig. 1, Participant Flow Diagram). Participants were recruited through university sports and performing arts clubs via email communication to the head coach or teacher, and Facebook adverts to targeted athletes. Inclusion criteria included no (or little) prior experience of flow education, a level of competence in one’s focal activity to ensure skill acquisition did not disrupt the activity experience, and a minimum age of 18 years. Participants were provided with an information letter outlining their rights as a participant and provided their informed consent prior to study commencement. The 26 participants (5 male, 21 female) were aged between 18 and 51 years old (*M* = 34). Participants engaged in a range of activities for the study including dance training (*n* = 15), dance performances (*n* = 6), kitesurfing (*n* = 1), gym routines (*n* = 1), bicycle spin classes (*n* = 1), singing (*n* = 1), and barista activity (*n* = 1). A small number of these participants (*n* = 4) engaged in the program entirely online, whilst a larger group (*n* = 22) received in-person training.

#### Intervention Description

The flow workshop was split into four sections—flow concept, flow antecedents, experiential dimensions, and practical tips from prior applied flow training studies. The intervention focused on an education of flow unspecific to a specific domain as flow has been researched to be consistent across domains. Specifically, an individual’s metacognition surrounding flow and personal ability to self-lead and self-regulate towards flow was targeted.

In section one, printed worksheets and a 40-minute discussion on the *concept* of flow included an initial identification of the day-to-day variability of personal experience, including a practical breath-holding activity to invoke an immediate stress response and highlight shifts in one’s subjective experience. Additionally, the experience of flow was then described through outlining anecdotal flow experiences, outlining the outcomes associated with flow, detailing the three experiential dimensions of flow (absorption, effort-less control, intrinsic reward) as outlined by Norsworthy et al. (2021), and a final discussion on personal flow experiences thought to be experienced by the participants.

In section two, Norsworthy et al.’s (2021) *antecedents* to flow and their sub-themes were described, and then practically experienced through a challenging task. Aspects relating to the antecedent ‘optimal level of challenge’ and the sub-themes ‘clear task goals’, ‘immediate and unambiguous feedback’, and ‘building self-efficacy’, were first described, then experienced by manipulating the clarity of task goals, feedback, and perceived challenge levels during a workshop game involving keeping an increasing number of balloons in the air (both individually and in a group) using any body part—the difficulty increased as the number of balloons increased. The participants’ experience of the challenging task was then discussed for approximately 40 min in small groups for practical relevance. Aspects of a ‘high motivation’ were discussed within the group and between participants, outlining how high motivation has occurred for participants in the past and an overview of how intrinsic motivation, extrinsic motivation, interest, subjective value, and the importance placed on the task can affect motivational force. The experience of changing the level of difficulty and motivational force was then discussed in teams of two or three in which each individual shared examples of how the learning could be practically relevant to their personal flow activity chosen to do alongside the intervention. Participants were then asked to reflect on these antecedents, the mindset that they typically applied to their chosen flow activity prior to the intervention, and whether their current mindset was conducive to flow or not.

In section three, participants were given practical tips to target the core *experiential flow dimensions* of ‘absorption’ and ‘effort-less control’, lasting approximately 40 min. The dimension ‘absorption’ was initially detailed and then targeted through practicing sustained attention on an easy-to-achieve task, and managing attention (visually and cognitively) when distractions occurred (also see Aherne et al., 2011; Cathcart et al., 2014); the logic being that managing attention towards more relevant information (of the task) positively impacts decision-making, the ability to accurately manage an ‘optimal challenge’ level, and self-efficacy (Pineau et al., 2014). ‘Effort-less control’ was then described and targeted through practising movement in a more effortless manner (than normal) during a challenging task; the logic being that through increased awareness of moving effortlessly, we learn to self-regulate cognitive and physiological processes towards a more effort-less sense of control (Gardner & Moore, 2007). ‘Intrinsic reward’ was then described and targeted through practising being attracted to the task (i.e., positive valence) and increasing arousal levels to be optimally aroused.

Lastly, section four of the workshop included helping participants integrate flow into their chosen activities for the study, lasting approximately 40 min. This section included a brief overview of *practical recommendations* from previous applied flow studies such as: not to make flow a continuous conscious outcome during task engagement; not to inhibit confidence by engaging in over challenging situations; not to increase extrinsic motivations only; not to confuse effortlessness with laziness or passiveness (see Csikszentmihalyi, 1975; Norsworthy et al., 2017a, b). In this section participants recorded key personal takeaways, personalised a single-page summary of the training content, and developed a personal action list to integrate the training into the preparation period of their upcoming activity sessions.

The two question-and-answer sessions were participant-led involving responding to raised questions regarding any ambiguity within the training, reconciling domain-specific examples of practical integration, and exploring their successes and failures.

### Assessment of Intervention Feasibility

#### Activity Competence

The pre-intervention survey (baseline) included seven items to assess participants’ activity competence level (e.g., “Please indicate your skill level for your activity”, with responses labelled, beginner, intermediate, advanced, amateur professional, professional), activity efficacy (e.g., “Do you feel you have the fundamental skills to perform in your activity?“), the extent of any previous mental skills training (e.g., “Have you received any prior mental skills training?“), the extent of any previous flow training (e.g., “Have you received any training on flow before?“), and whether any technical changes may interfere with the training (e.g., “Are you currently making many (or significant) changes in how you *technically* perform in your activity?“). All items were scored on a 7-point response scale ranging from 1 (*none/not at all*) to 7 (*extensive/absolutely*). See S1, Tables 1 and 2 for item details.

#### Participant Mindset

The pre-intervention survey included seven individual items that examined the participants’ perception of mental skills and their current mental state of application towards their flow activity (e.g., “Do you believe mental skills training can make a difference to your engagement in the activity?“ and motivation levels (e.g., “Are you currently motivated to engage in your activity?“). All items were scored on a 7-point response scale ranging from 1 (*not at all*) to 7 (a*bsolutely*). See S1, Table 3 for item details.

#### Workshop Perceptions

In the post-workshop survey, participants completed nine individual questions (not based on validated scales) assessing their perceptions regarding the workshop—three individual items derived by the authors assessed training fidelity (e.g., “The training was carried out and delivered professionally”), three items assessed the perceived usefulness of the training (e.g., “I felt the training was valuable”), and three items assessed participant engagement (e.g., “I was able to engage in the training to the best of my ability”). These 9 items were scored on a 7-point response scale ranging from 1 (*not at all true*) to 7 (*very true*), with higher scores corresponding to more positive evaluations (See S1, Table 4).

#### Perceptions of Program Components

After the program (i.e., 10 days following workshop participation), participants were presented with a questionnaire focused on their perceptions regarding the usefulness of the training components. This included individual questions regarding the usefulness of (a) understanding the *concept* of flow; (b) learning how to target the *antecedents* to flow; (c) learning how to target the *experience* of flow; (d) understanding how to *integrate* the training into their specific activity; and (e) the usefulness of the question-and-answer sessions. All items were scored on a 7-point response scale ranging from 1 (*not at all true*) to 7 (v*ery true*), with higher scores corresponding to more positive evaluations (See S1, Tables 5 and 6). In addition to pre-intervention measures, participants were asked whether the training on flow had an impact on the belief that mental skills training can make a difference to activity engagement (e.g., “Do you believe mental skills training can make a difference to your engagement?“), and the belief that the flow training will make a big difference (e.g., “Do you believe this flow training will make a big difference?“). Lastly 3 separate questions explored intervention improvements (e.g., “What would you change about the training? Please be specific.”). Participants were given a text field to write their answers (See S1, Tables 7, 8, and 9).

### Assessment of Preliminary Efficacy

#### Flow Scores

The Psychological Flow Scale (PFS; Norsworthy et al., 2021) was chosen to measure flow because it targets the three core experiential dimensions of flow which are not domain specific or dependent on nuanced experiential descriptions (such as the dimension ‘time transformation’) that are commonly measured but not always apparent in the flow literature, nor statistically robust when measured in the nine-dimensional model. Further, it does not conflate antecedents or pre-conditions within the measurement, utilises accessible language and dimensional definitions that can be comparable across domains and scientific disciplines, and measures a core experience of flow parsimoniously. Accordingly, participants were given the PFS at pre- and post-intervention to measure flow. The scale consists of 15 items, with five items for each of the three flow dimensions (absorption, effort-less control, intrinsic reward). All items were scored on a 7-point response scale ranging from 1 (*strongly disagree*) to 7 (*strongly agree*). Global flow scores were determined using an average of all 15 items. Subscale scores (i.e., absorption, effort-less control, intrinsic reward) were determined by averaging responses to the five items in each subscale.

Participants completed the scale based on their most intense experience whilst participating in their last three flow activity sessions outside of the educational workshop (inherent within the PFS questioning). Focusing on a specific experience as opposed to an aggregated overview of multiple events or aggregated overview of experiences within a single event, was deemed important to ensure answers did not conflate multiple experiences. Participants were encouraged to answer the PFS questionnaire as soon as possible after the last flow activity.Norsworthy et al. (2021) reported reliability scores for the global factor (0.895) and subscales (absorption = 0.928; effort-less control = 0.875; intrinsic reward = 0.937) and found preliminary support (through bi-factor modelling) for a domain-general measure of flow. Cronbach alpha values for pre-intervention PFS scores (*n* = 26) were 0.981 for the global score, 0.972 for absorption, 0.963 for effort-less control, and 0.976 for intrinsic reward. These values for post-intervention PFS scores (*n* = 26) were 0.967, 0.965, 0.947, and 0.955 respectively.

#### General Program Outcomes

Participants were asked to report (at 10 days after workshop) on six general outcomes. Specifically, whether the training was effective in improving their ability to (a) increase the intensity of flow states, (b) increase the frequency of flow states, (c) feel more confident that they have the necessary skills to find flow in their activity, (d) feel more confident in becoming highly focused and absorbed into their activity, (e) feel more confident to feel an effortless sense of control in their activity, and (f) feel more confident to enjoy their activity (See S1, Table 8). Participants were also asked direct single-item questions on whether the program reduced stress (e.g., “The training reduced my stress in daily life beyond my activity”), improved confidence in handling stressful situations (e.g., “The training helped me feel more confident in handling stressful situations?“), improved performance (e.g., “The training helped improve my performance”), improved ability to enjoy their activity (e.g., “The training helped me enjoy my activity more”), could be applied to their activity (e.g., “I feel that flow training can be applied to my activity”), and improved confidence in performance (e.g., “The training helped me feel more confident in my performance”). All items were scored on a 7-point response scale ranging from 1 (n*ot at all true*) to 7 (v*ery true*), with higher scores corresponding to more positive evaluations. See S1, Table 10 for item details. Participants were also (qualitatively) asked to describe the most *effective* aspects of the program, the most *ineffective* aspects of the program, list of training benefits, and whether the training simply made them more aware of flow or actually made a difference to finding flow more frequently or intensely (See S1, Tables 11, 12, 13 and 14). In addition to the PFS, participants were given direct questions (pre- and post-intervention) targeting flow experience characteristics (entry, duration, occurrence, intensity; see S2).

#### Flow ‘Outcomes’

Participants were asked pre- and post-intervention to report self-rated performance, well-being, intrinsic motivation, choice/autonomy, competence, and stress related to their chosen life activity. To examine *self-rated performance* scores, a single self-report item was used to examine perceived performance. Participants responded on a response scale from 1 (*very low performance*) to 11 (*very high performance*) to the item, “Please rate how you felt you performed in your chosen activity”. To assess *well-being*, the WHO-5 scale (see Topp et al., 2015) was utilised, which consists of 5 items (e.g., “I have felt active and vigorous”) scored on a response scale from 1 (*at no time*) to 7 (*all of the time*). As recommended, all 5 items were summed to determine a well-being score (Cronbach’s alpha = 0.885 for pre-intervention and 0.830 for post-intervention).

The multidimensional 22-item Intrinsic Motivation Inventory (IMI; Ryan, 1982) was used to measure *choice* (autonomy; example item: “I felt that it was my choice to do the task”; Cronbach’s alpha for subscale score = 0.833 pre-intervention and 0.720 post-intervention); *intrinsic motivation* (example item: “I found the task very interesting”, Cronbach’s alpha = 0.919 pre-intervention and 0.913 post-intervention); *competence* (example item: “I think I am pretty good at this task”, Cronbach’s alpha = 0.815 pre-intervention and 0.869 post-intervention); and *stress* (pressure/tension; example item:, “I felt tense while doing the task”; Cronbach’s alpha = 0.804 pre-intervention and 0.687 post-intervention). Items from each subscale were averaged to determine subscale scores. Single-item measures were used to examine participants’ self-reported *ability to handle stress* (e.g., “I felt capable of handling stress”), felt *stress* (e.g., “I felt very stressed”), levels of *anxiety* (e.g., “I felt anxious / worried”), *ability to meet challenges* (e.g., “I felt that I could meet the challenge”), *ability to feel excited about the challenge* (e.g., “I felt excited about the challenge”), *motivation* levels (e.g., “I am very motivated to play”), *desire to learn* and grow (e.g., “My desire to learn and grow as an athlete was very high”), and a sense of *curiosity* towards the performance (e.g., “I perceived my performance with curiosity”). Responses were provided using a response scale ranging from 1 (*not at all true*) to 7 (*very true*).

#### Data Analysis

Data were initially screened for missing values. The few (< 3%) missing data items (existing in single-item measures only) were not replaced. Descriptive data were generated to illustrate aspects of recruitment, participant perspectives, and certain post-intervention responses to the preliminary assessments of outcomes that were not measured pre-intervention. Given that studies of this nature are not designed based on power or necessary sample size estimations, to examine change in outcome variables from pre-to-post-intervention, standardised effect sizes (Cohen’s *d*) were calculated to indicate the magnitude of any change across the program. A sensitivity analysis (Perugini et al., 2018; conducted using G*power, with power = 0.80 and alpha = 0.05) indicated that the minimal effect size of 0.57 could be detected with a sample of 26, and 0.50 with a sample of 34. For the interested reader, paired samples t-tests results are displayed in Table 1. IBM SPSS (V27) were used for all quantitative analysis. To examine participants’ qualitative reports, a thematic content analysis (TCA) approach using Anderson’s (2019) TCA protocol was conducted by the authors in order to identify common categories. Any disagreements were resolved through open discussion between the authors whilst revisiting source data to ensure integrity of the participants’ meaning.

## Results

### Feasibility Components

#### Activity Competence

In total, 26 participants were deemed to have the necessary skills (although some self-reported as being beginners) to engage in their activity without technical interruptions (*M* = 3.00; *SD* = 1.27) in their activity, had the fundamental skills to perform the activity (*M* = 5.04; *SD* = 1.77), felt moderately confident in their ability to perform in their activity (*M* = 4.96; *SD* = 1.37), felt moderately mentally strong in their activity (*M* = 4.50; *SD* = 1.32), and had minimal (or no) prior flow training experience (*M* = 1.12; *SD* = 0.44). See S1, Tables 1 and 2.

#### Participant Mindset

Participants’ existing application towards their activity was deemed not to be problematic to program engagement. Participants reported (pre-intervention) that they believed mental skills training could make a difference to their engagement (*M* = 5.80; *SD* = 1.53) and performance (*M* = 6.08; *SD* = 1.80) in their activity, were motivated to engage in their activity (*M* = 5.25; *SD* = 1.45), and moderately believed that flow training could make a big difference (*M* = 5.40; *SD* = 1.15). See S1, Table 3.

#### Workshop Perceptions

Post-workshop (initial 3-hour training) survey results indicated high *fidelity of training* scores (*M* = 6.34; *SD* = 1.15). On a 1-to-7 response scale, participants reported that the training was engaging (*M* = 6.00; *SD* = 0.98), understandable (*M* = 6.38; *SD* = 1.87), and delivered professionally (*M* = 6.63; *SD* = 0.61). *Usefulness of training* scores were high (*M* = 5.98; *SD* = 1.04), with participants reporting that the training was practical (*M* = 5.69; *SD* = 1.15), valuable (*M* = 6.16; *SD* = 0.92), and useful (*M* = 6.09; *SD* = 1.06). Participant engagement scores were high (*M* = 6.09; *SD* = 1.07), with participants reporting that they were undistracted (*M* = 6.09; *SD* = 0.93), came to the training with an open mind that flow training could work (*M* = 6.13; *SD* = 1.31), and were able to engage to the best of their ability (*M* = 6.06; *SD* = 0.98). See Table 4.

#### Perceptions of Program Components

Post-intervention survey results indicated that the majority of participants reported the overall program was useful (*M* = 5.50; *SD* = 1.36). Specifically, the training on the flow concept (*M* = 5.58; *SD* = 1.47), targeting antecedents (*M* = 5.35; *SD* = 1.29), targeting experiential dimensions (*M* = 5.27; *SD* = 1.46), integrating the training into their activity (*M* = 5.15; *SD* = 1.38), and question and answer sessions (*M* = 5.31; *SD* = 1.59) were all considered helpful for increasing flow intensity. Participants were split when ranking which of the above components to be more or less helpful, though mean scores suggested that targeting the antecedents of flow was slightly (on average) more helpful (see S1, Table 9). Qualitative analysis indicated a wide variety of useful program components, with the most popular components being the idea of ‘preparing the mindset’ to get into a flow state before commencing an activity, the opportunity to practice the flow skills in the workshop during a task, and the takeaway material (see S1, Table 7). In terms of training improvements, the largest reported theme was that “the training didn’t require any changes”, whilst a number of participants asked for more time to engage in the practical tasks (See S1, Tables 6 and 7).

### Assessment of Preliminary Efficacy

#### Flow Scores

In examining the difference between pre- and post-intervention scores, global flow scores based on their activity experience (*M* pre = 4.66; *M* post = 5.55) scores increased by 18.02% (*d* = 0.84, *p* < .001), absorption scores (*M* pre = 4.56; *M* post = 5.46) increased by 19.74% (*d* = 0.71, *p* < .01), effort-less control scores (*M* pre = 4.23; *M* post = 5.38) increased by 27.19% (*d* = 0.83, *p* < .001), and intrinsic reward scores (*M* pre = 5.20; *M* post = 5.79) increased by 10.96% (*d* = 0.54, *p* < .05). See Table 1 for detailed statistics.

#### General Program Outcomes

Participants reported that the program was effective in improving their ability to increase the intensity (*M* = 5.69; *SD* = 1.26) and frequency (*M* = 5.77; *SD* = 1.14) of flow states. Participants reported that the training caused them to feel confident that they had the necessary skills to find flow (*M* = 5.46; *SD* = 1.36), confident in their ability to become highly focused and absorbed (*M* = 5.46 *SD* = 1.36), feel an effortless sense of control (*M* = 5.31; *SD* = 1.35), and enjoy their activity (*M* = 5.35; *SD* = 1.29). See S1, Table 8 for detailed statistics. There were strong perceptions (also see S1, Table 10) that the overall program reduced stress (*M* = 4.31; *SD* = 1.26), improved confidence in handling stressful situations (*M* = 4.69; *SD* = 1.32), improved performance (*M* = 5.00; *SD* = 1.33), improved ability to enjoy their activity (*M* = 5.15; *SD* = 1.35), could be applied to their activity (*M* = 5.77; *SD* = 1.45), improved confidence in performance (*M* = 5.27; *SD* = 1.31), and improved confidence in applied flow skills (*M* = 5.46; *SD* = 1.31).

Qualitative analysis indicated that participants varied in their preference regarding which aspect of the intervention program was most effective and ineffective to finding flow (See S1, Tables 11 and 12). Equally, participants described a variety of benefits deriving from the training; the majority surrounding a theme of increased confidence and competence, a better ability to prepare their mindset, increased motivation, and improved self-trust (See S1, Table 13). When asked whether the training simply made them more aware of flow or actually helped them to find flow, the majority (54%) of participants reported, “both” (see S1, Table 14). For the interested reader, results for the preliminary efficacy of measuring flow entry, duration, occurrence, and intensity can be found at S1, Table 15.

#### Flow Outcomes

We recognise that the study was not designed to be powered to detect (statistical) lasting effects over time; however, we present the output from paired sample t-tests, including effect sizes, and associated *p* values and confidence intervals, for the interested reader below. See Table 1, and S1, Table 16, for detailed statistics regarding the preliminary assessment of outcomes.

##### Self-Rated Performance

Participants self-reported a mean performance score of 4.19 (*SD* = 1.44) pre-intervention and 5.23 (*SD* = 0.95) post-intervention, increasing by a large effect size (*d* = 0.81, *p* < .001) and 24.82%. Total (pre- and post-intervention) performance scores positively correlated with total PFS scores (*r* = .727, *p* < .001). See S1, Table 16.

##### Well-Being

Participants self-reported a mean well-being score of 88.15 (*SD* = 22.81) pre-intervention and 105.85 (*SD* = 20.99) post-intervention, increasing by a moderate to strong effect size (*d* = 0.68, *p* < .01) and 15.53%. Total well-being scores positively correlated with total PFS scores (*r* = .673, *p* < .001).

##### Choice/Autonomy

Participants self-reported a mean choice/autonomy score of 5.2 (*SD* = 1.10) pre-intervention and 5.58 (*SD* = 1.29) post-intervention, increasing by a low effect size (*d* = 0.38, *p* = .066) and 4.56%. Total choice/autonomy scores positively correlated with total PFS scores (*r* = .534, *p* < .001).

##### Intrinsic Motivation

Participants self-reported a mean score of 4.9 (*SD* = 1.28) pre-intervention and 5.48 (*SD* = 1.13) post-intervention, increasing by a large effect size (*d* = 0.72, *p* < .01) and 11.63%. Total choice scores positively correlated with total PFS scores (*r* = .534, *p* < .001).

##### Competence

Participants self-reported a mean competence score of 4.32 (*SD* = 1.17) pre-intervention and 5.22 (*SD* = 0.90) post-intervention, increasing by a large effect size (*d* = 0.96, *p* < .001) and 11.63%. Total competence scores positively correlated with total PFS scores (*r* = .729, *p* < .001).

##### Stress

Participants self-reported a mean stress score of 3.89 (*SD* = 1.23) pre-intervention and 2.75 (*SD* = 1.10) post-intervention, decreasing by a large effect size (*d* = -1.08, *p* < .001) and 29.46%. Total stress scores negatively correlated with total PFS scores (*r* = − .372, *p* < .01).

##### Single-Item Measures

Large effect sizes were reported for *ability to handle stress* (+ 25%). Medium effect sizes were reported for *felt stress* (-33%) and felt anxiety (-49%). Small effect sizes were reported for *ability to meet the challenge* score (+ 13%), *ability to feel excited about the challenge* (+ 1%), *motivation level* (+ 8%), *desire to learn and grow* (+ 6%), and *curiosity* (+ 9%). See Appendix, Table 1.

##### Preliminary Assessment of Online vs In-Person Flow Training

For the interested reader, results examining the difference between online and in-person training can be found in S1, Table 17, and S2.

## Discussion

Positive psychology interventions have been increasingly used to affect performance (Swann et al., 2012) and psychological well-being (Bolier et al., 2013). Despite applied flow studies appearing across sciences, no ‘gold standard’ intervention or well-designed model for designing an effective intervention to promote flow exists (Norsworthy et al., 2017b). We conducted a single-group, non-randomized feasibility trial, and produced a detailed account of a new educational flow training program to develop a solid research foundation for intervention “curriculum” and quality standards, and for measuring results. The feasibility of the flow training program was assessed by evaluating participant retention, perceptions about and experiences of the program, and perceptions about the flow education training. We aimed to provide insight into the preliminary efficacy of the program by measuring flow and its three inclusive dimensions (absorption, effort-less control, intrinsic reward; as outlined by Norsworthy et al., 2021), self-rated performance, well-being, intrinsic motivation, choice/autonomy, competence, and stress. In the material that follows, we consider feasibility and preliminary efficacy findings, and highlight study limitations and directions for future research.

### Program Feasibility

In general, our findings indicated that flow training is feasible through an educational intervention, was well received by program participants, and yielded positive preliminary effects. In regard to recruitment and retention, participant acquisition was successful using Facebook advertising and direct contact with local sporting clubs and performing arts centres. Clubs were initially difficult to recruit when targeting teams, so the participant recruitment strategy changed to contacting individuals. There was a 42% conversion rate from interested participants to program completion and participants regarded the workshop to be highly engaging, understandable, delivered in a professional manner, practical, valuable, and useful. This evidence suggests that the program is feasible to be positioned in a larger experimental study.

Participants were varied in their assessment of what was most useful in terms of program components. Targeting the antecedents of flow within the training gained the highest scores, though training on the flow concept, targeting experiential dimensions, integrating the training into their activity, and post-workshop question-and-answer sessions were all deemed of value. This suggests that it may be important to include all the above program components, as participants varied in the value they placed on the practical and theoretical aspects of the training. In terms of improving the program, the overall consensus from participants was that the training didn’t require changing; however, some participants indicated preference for more time to practice and implement practical tasks. Recommendations for future educational interventions on flow therefore include longer (i.e., full day or two half-day) educational sessions to allow further time and discussion around the practical tasks within the training. Unexpectedly, the integration component of the training—that delivered both practical tips and time to think about how to integrate the training into the individual’s activity—received lower scores of usefulness than all other components (on average). It is possible that these findings might change given more time to detail how to integrate this training within same-activity-groups, though further research with bigger samples is required.

### Program Efficacy

Evidence of the feasibility of the program is important, but it is also important to understand the value and outcomes of the intervention. The design of the study (i.e., absence of a control arm; small sample size) mean any efficacy findings are preliminary and any conclusion made of the efficacy data in this study should be taken with caution. Nevertheless, the findings of this study were favourable to program efficacy.

Participant responses were positive with respect to their experiences of the program. Participants reported feeling more confident that they had the necessary skills to experience flow, and that the flow training was effective in improving participants’ ability to increase the intensity and frequency of flow. Further, participants reported that they felt more confident in their ability to become highly focused and absorbed, feel an effortless sense of control, and enjoy their activity. Participants self-reported that the overall program could be applied to their activity, and reported improved confidence in handling stressful situations, performance, ability to enjoy their activity, confidence in finding flow, and reduced stress. Participants’ qualitative reports also indicated that the program better equipped them to prepare their mindset, increase motivation, and improve self-trust. Lastly, the majority of participants reported that the training made them both more aware of flow in their life, and actually helped them to find flow more intensely and frequently.

On average, participants increased global flow scores (18.02%), absorption (19.74%), effort-less control (27.19%), and intrinsic reward scores (10.96%), and self-reported to increase their flow-entry (262%) and intensity (10.41%) of flow, suggesting that the intervention may be effective for increasing flow. Total flow intensity scores correlated with total PFS scores suggesting that PFS scores may align with self-reported intensities of simple descriptions of flow, and although inter- and intra-differences will exist, these preliminary results suggest that higher PFS scores may indicate higher self-reported flow intensities. In regard to other outcomes, self-reported single-item measures and statistically significant scores revealed an increase in performance, confidence, competence, well-being, intrinsic motivation, and choice/autonomy, and a decrease in stress. The strongest outcome and effect sizes included an increase in self-rated performance (24.82%) and ability to handle stress (24.82%), and a reduction in felt stress (33.01%) and anxiety (48.89%). These results are consistent with prior research in which flow experiences have been aligned with positive development and high functioning (also see Flett, 2015; Klasen et al., 2012; Norsworthy et al., 2021; & Swann et al., 2017) and as a buffer or coping mechanism to anxiety and stress (also see Llorens & Salanova, 2017; Peifer et al., 2014; Sadlo, 2016; Waples & Knight, 2017). Further causal experimental research accounting for inter- and intra-individual differences is required. Preliminary data suggests that online flow training should not be overlooked in favour of in-person training, though more data is required.

### Limitations, Future Directions, and Conclusions

Although single-arm, non-randomized designs are acceptable and well-recognised for feasibility trials (Lancaster & Thabane, 2019), the lack of a control-arm and a small sample size does preclude causal conclusions regarding the effectiveness of the program. Demand characteristics of the study such as an expectation to improve flow from flow training may be a limitation, therefore future randomized control studies are recommended. A valuable extension of this work, therefore, would be an experimental approach that includes a control group and a larger sample that includes a more balanced gender count. It is recommended that in future studies, multiple baseline and post-workshop data points (especially regarding outcomes), temporal retention data, and qualitative follow-up data are collected to assess the sustainability of any effects. It must be noted that the current study was applied to individuals as opposed to teams. Applied literature for flow in teams differs from individual application due to the complications of individual goals and collective goals (Van den Hout et al., 2018), and would need to be taken into consideration when designing a flow training educational program for groups. This study’s inclusion criteria included participants that were relatively competent in their activity—so that technical improvements would not disrupt measures; future studies involving participants learning a new activity are required to identify whether flow training can accelerate the learning process. The use of retrospective subjective reports to measure flow has previously been questioned (Norsworthy et al., 2021); therefore, at such a time when non-retrospective physiological measures become more refined, it is suggested that these measures be used in conjunction with PFS measures. Additionally, a limitation of the present study was that the authors were not present during data collection, and as such, a level of trust towards procedural compliance (by participants) was required. For instance, a 1-page document outlining the participants’ key takeaways and applied skills was co-created with the participants at the end of the workshop to help facilitate future activity preparations; this study did not, however, specifically address adherence to the preparation rituals (i.e., checking the 1-pager). Having a more robust evaluation process on the adherence and application of the training would help to ensure that training takeaways (such as preparation routines) are applied. Further measures on procedural adherence (such text reminders, preparation check-ins, and requests for participant assurances), for example, could be deployed for greater clarity.

In conclusion, our study achieved the objective of evaluating the feasibility and preliminary efficacy of an educational flow training program. This study provides a detailed account of what constitutes a flow training program, and evidence indicating preliminary efficacy of the program in terms of increasing flow, performance, confidence, well-being, intrinsic motivation, autonomy, and a reduction in, and ability to handle, stress and anxiety. The results and positive participant perceptions of the program suggest the study’s adopted intervention is a positive starting point to developing an effective flow training program. The study adds detailed information and preliminary insights to an otherwise scant area of research and has established a foundation (and further suggestions) for the implementation of a larger scale program. The training employed is elementary and non-causal, and any practitioners seeking to apply the contents of this study are encouraged to follow the guidelines mentioned within. In an applied setting these findings and the ability to potentially self-generate the experience of flow with greater intensity is exciting.

### Supplementary Information

Below is the link to the electronic supplementary material.

ESM 1 (DOCX 55.0 KB)

ESM 2 (DOCX 19.3 KB)

## Appendix 1

**Table 1:** Pre-to-post intervention scores for preliminary flow and outcome scores

					95% Confidence Interval of the Difference		Paired Sample T-Test			Cohen’s
	*M pre (SD)*	*M post (SD)*	*M diff* (*SD*)	% diff	Lower	Upper	*t*	*df*	*p*	*d*
Flow (PFS) Measures										
Global Flow	4.66 (1.26)	5.55 (1.16)	0.89 (1.05)	18.02	0.45	1.31	4.27	25	< 0.001	0.84
Absorption	4.56 (1.46)	5.46 (1.19)	0.9 (1.26)	19.74	0.39	1.41	3.63	25	< 0.01	0.71
Effort-less Control	4.23 (1.33)	5.38 (1.13)	1.15 (1.39)	27.19	0.59	1.72	4.23	25	< 0.001	0.83
Intrinsic Reward	5.20 (1.38)	5.79 (1.38)	0.57 (1.10)	10.96	0.15	1.04	2.76	25	< 0.05	0.54
Outcomes (Using Scales)										
Performance	4.19 (1.44)	5.23 (0.95)	1.04 (1.28)	24.82	0.52	1.56	4.18	25	< 0.001	0.81
Well-being	88.15 (22.81)	101.85 (20.99)	13.69 (20.05)	15.53	5.59	21.79	3.48	25	< 0.01	0.68
Choice (Autonomy)	5.20 (1.1)	5.58 (1.29)	0.38 (1.02)	7.30	0.03	0.80	1.92	25	0.066	0.38
Intrinsic Motivation	4.90 (1.28)	5.48 (1.13)	0.57 (0.79)	11.63	0.25	0.89	3.69	25	< 0.01	0.72
Competence	4.32 (1.17)	5.22 (0.90)	0.91 (0.95)	21.06	0.53	1.29	4.90	25	< 0.001	0.96
Stress	3.89 (1.23)	2.75 (1.10)	-1.15 (1.053)	-29.46	-1.57	-0.72	-5.55	25	< 0.001	-1.08
Self-reported Outcomes										
Ability to Handle Stress	4.19 (1.41)	5.23 (0.91)	1.04 (1.40)	24.82	0.47	1.6	3.78	25	< 0.01	0.74
Felt Stress	3.15 (1.77)	2.12 (1.45)	-1.04 (1.90)	33.01	-1.81	-0.27	-2.78	25	< 0.05	− 0.54
Anxiety	3.54 (1.62)	2.00 (1.09)	-1.54 (1.79)	48.89	-2.26	-0.81	-4.37	25	< 0.001	− 0.86
Ability to Meet Challenges	4.81 (1.2)	5.42 (1.33)	0.62 (1.53)	12.89	0.00	1.23	2.06	25	0.050	0.40
Excited About Challenge	5.15 (1.62)	5.19 (1.47)	0.04 (1.51)	0.70	-0.57	0.65	0.13	25	0.898	0.03
Motivation Levels	5.04 (1.66)	5.42 (1.42)	0.38 (1.34)	7.54	-0.15	0.92	1.48	25	0.153	0.29
Desire to Learn and Grow	5.30 (1.72)	5.62 (1.55)	0.31 (1.32)	5.85	-0.23	0.84	1.19	25	0.246	0.23
Curiosity	4.88 (1.45)	5.31 (1.46)	0.42 (1.45)	8.61	-0.16	1.01	1.49	25	0.149	0.29

*Note.* All values (except *p* values) rounded to 2 decimal points. Negative differences for stress, felt stress, and anxiety all represent positive reduction in scores.
