# Supplementary material for: Psychological Flow Training: Feasibility and Preliminary Efficacy of an Educational Intervention on Flow
Source: Int J Appl Posit Psychol. 2023 May 23:1–24. Online ahead of print. doi: 10.1007/s41042-023-00098-2 (PMC10204032; doi:10.1007/s41042-023-00098-2)
Supplement: Supplementary file 1 — (DOCX 55.0 KB) [file 41042_2023_98_MOESM1_ESM.docx]

**Supplementary Material**

**for**

**Flow Training: feasibility trial and preliminary efficacy of an educational intervention on flow**

**Supplementary Table 1.** Participants’ perception of their activity competence

| Item | *N* | % |
| --- | --- | --- |
| Beginner | 6 | 23 |
| Intermediate | 9 | 35 |
| Advanced | 7 | 27 |
| Amateur Professional | 1 | 4 |
| Professional | 3 | 11 |

**Supplementary Table 2.** Participants’ prior training

| Item | *N* | Mean (*SD*) |
| --- | --- | --- |
| Have you received any prior mental skills training? | 26 | 2.63 (1.50) |
| Have you received any training on 'flow' before? | 26 | 1.12 (0.44) |
| Are you currently making many (or significant) changes in how you technically perform in your activity? | 26 | 3.00 (1.27) |

Note: Items were scored 1-7, where higher scores denote more extensive training or changes.

**Supplementary Table 3.** Participants’ perceptions of the program (pre-intervention)

| Item | *N* | Mean (*SD*) | Percentage of scores above 70% |
| --- | --- | --- | --- |
| Do you feel you have the fundamental skills to perform in your sport/activity? | 26 | 5.04 (1.77) | 68% |
| Do you believe mental skills training can make a difference to your engagement in the sport? | 26 | 5.80 (1.53) | 84% |
| Do you believe mental skills training can make a difference to your performance in the sport? | 26 | 6.08 (1.08) | 96% |
| Are you currently motivated to engage in your sport/activity? | 26 | 5.25 (1.45) | 72% |
| Do you feel confident in your ability to perform in your sport/activity? | 26 | 4.96 (1.37) | 72% |
| Do you feel mentally strong in your sport/activity? | 26 | 4.50 (1.32) | 52% |
| Do you believe this flow training will make a big difference? | 26 | 5.40 (1.15) | 77% |

Note: Items were scored 1-7, where higher scores denote more positive perceptions.

**Supplementary Table 4.** Participants’ perceptions of the training

| Item | N | Mean (SD) | Percentage of scores above 70% |
| --- | --- | --- | --- |
| I felt the training was engaging | 34 | 6.00 (0.98) | 94% |
| I felt the training was practical | 34 | 5.69 (1.15) | 85% |
| I will use this training | 34 | 6.09 (1.06) | 94% |
| I participated in the training with an open mind that flow training could work | 34 | 6.13 (1.31) | 94% |
| The training was carried out and delivered professionally | 34 | 6.63 (0.61) | 100% |
| I felt the training was valuable | 34 | 6.16 (0.92) | 94% |
| I was able to understand the training content | 34 | 6.38 (1.87) | 97% |
| I was undistracted by the other people in the training and able to focus on the training | 34 | 6.09 (0.93) | 94% |
| I was able to engage in the training to the best of my ability | 34 | 6.06 (0.98) | 91% |

Note: Items were scored 1-7, where higher scores denote more positive perceptions.

**Supplementary Table 5.** Participants’ perceptions on program effectiveness

| Item | N | Mean (SD) | Percentage of scores above 70% |
| --- | --- | --- | --- |
| I feel that flow training can increase the *intensity* of flow states | 26 | 5.69 (1.26) | 77% |
| I feel that flow training can increase the *frequency* of flow states | 26 | 5.77 (1.14) | 88% |
| I felt the training was very useful | 26 | 5.50 (1.36) | 77% |
| Because of the training I feel more confident that I have the necessary skills to find flow in my activity | 26 | 5.46 (1.36) | 81% |
| Because of the training I feel more confident in becoming highly focused and absorbed into my activity | 26 | 5.46 (1.36) | 85% |
| Because of the training I feel more confident to feel an effortless sense of control in my activity | 26 | 5.31 (1.35) | 88% |
| Because of the training I feel more confident to enjoy my activity | 26 | 5.35 (1.29) | 81% |
| Training on understanding the concept of Flow and recognising it in my life was helpful to finding more flow in my activity | 26 | 5.58 (1.47) | 85% |
| Training on creating the antecedents (Challenge Your Capacity and Increase Motivation) was helpful to finding more flow in my activity | 26 | 5.35 (1.29) | 77% |
| Training on recreating the experience of flow (Absorption-focused attention –/ Effortlessness in action) was helpful to finding more flow in my activity | 26 | 5.27 (1.46) | 73% |
| Training that focused on integrating the training into my specific activity was helpful to finding more flow in my activity | 26 | 5.15 (1.38) | 81% |
| Do you believe the Q&A sessions (post initial training) were very helpful? | 26 | 5.31 (1.59) | 73% |
| Do you believe mental skills training can make a difference to your engagement? | 26 | 6.50 (0.81) | 100% |
| Do you believe this flow training will make a big difference? | 26 | 5.42 (1.17) | 73% |

Note: Items were scored 1-7, where higher scores denote more positive perceptions.

**Supplementary Table 6.** Participants’ ranking of the effectiveness of program stages

|  | Conceptual understanding of flow | Applied training to target the antecedents | Applied training to target the experience | Integration training to specific activity and context |
| --- | --- | --- | --- | --- |
| First | 7 | 6 | 6 | 5 |
| Second | 7 | 11 | 5 | 4 |
| Third | 4 | 6 | 11 | 6 |
| Fourth | 7 | 2 | 3 | 10 |
| Overall score* | 65 | 71 | 64 | 54 |

*Overall score was calculated based on 4 points for 1^st^, 3 points for 2^nd^, 2 points for 3^rd^, and 1 point for 4^th^ place. Higher scores represent more effective training.

**Supplementary Table 7.** Participants’ responses to training usefulness

| Question: What was most useful from the training? |
| --- |
| - Learning to focus on the process of my tasks, not only the outcome. - Receiving strategies as to how to maximise flow and harness it in everyday life - The confidence map - I feel it was actually the balloon activity, not only understanding other people in the group and working together but also just focusing properly on the task at hand, and understanding how “practice” can aid in it running more smoothly - Reinforcing prior knowledge and the relevance to my activity was motivating - Overall, very good - Liked the takeaway cheat sheet to make it practical - It was great being able to immediately relate what you were learning to your chosen activity by completing written exercises throughout and at the end of each section. Also, the practical balloon activity was useful as I could feel the effects of flow in action. - Facts that I could take away to demonstrate or prove how flow is actually effective. - Explanation of brain mechanics, flow, and techniques - I liked the motivation flow chart, its practical and easy to understand - Strategies to get in the flow, minimise barriers and shape experiences - The idea of 'preparing' the mindset to get into a flow state before commencing an activity. - I direct negative thoughts into different directions - The tools for improve your confidence and the explanation about intrinsic and extrinsic - The verbal explanation - Being able to apply flow to a situation with the balloon activity. Being given vocabulary tools surrounding the concept of flow in order to dictate the feeling verbally and mentally. - Being in flow is focussing on enjoying the experience - Giving examples of entering flow state so that we could relate it to our personal circumstances. The training was well-suited to accommodate a range of sports and performance areas - The booklet with the summarised information and the easily understandable format/structure of the session which broke sections up into smaller chunks. - Solidified my understanding of flow and how to achieve it. It was good to hear that what I had been implementing was proven to be effective. - Methods to promote flow state. (Before conducting activity) - Learning the difference between Flow and Mental Focus - The balloon game to put the theory into practice - Showing my how short my attention was when looking at the flame. How much I need to practice on focusing. - it’s good to know how flow work for me - The specific examples given - "Skill wall" - Learning what flow is and the best ways to get there. - Definitely the 3 key aspects of flow and the segmented sections of it - It was excellent encouraging us to embrace stress as a positive - Understanding and breakdown of the concept - The balloon game was a practical way to demonstrate and show how flow changes - The idea to aim for flow state and not a certain outcome - Strategies - Reframing a situation and focusing on entering a state of flow through motivation. - The intrinsic and extrinsic motivation and the tools to improve confidence - Focus on enjoying the flow experience - The structure of the course as it was easy to follow. Each aspect of the training followed on to the next - Pre activity prep - Hearing other participants experiences - Learning that you can reframe your experience. - to use flow technique into my strength training as well as my work - Analysing before and after the practical exercises |

**Supplementary Table 8.** Participants’ responses to possible changes to the training

| Question: What would you change about the training? |
| --- |
| - More time to hear everyone’s answers to what we have written down. Gives insight into their thoughts and could spark more of our own. - I would not change anything I feel that the entirety of the session was well planned and as a result I have come out of it feeling motivated to further my interests in flow - can't think of anything - it was really good. Perhaps just slightly too long (30min shorter would be perfect for me) - I think mentioning a bit about how much training you need to be very competent in an activity, expand on maybe the 10,000-hour theory and tie them together - More explicit links to theory - Make it a little more step by step some of the activities were a little unclear - it is all a matter of time really, but I found that the sharing talking time with partners was a little one sided, maybe a point in time where you call to swap people who are talking so both people get examples? - Near the end i just lost focus because 3 hours is a long time to stay focused - Time allotted to discuss personal stories/preferences. It wasn’t enough at times. - N/a - PowerPoint more descriptive because I am not a native speaker. Handout on key point still good - I would make It more practical with exercises or practical examples that we can use in a daily basis - The handouts - Some more work on factors that influence the ability to drop into flow state, e.g., self-confidence etc. - More practical examples that demonstrate application of the flow theory. - Making the training more specific. Perhaps having follow up sessions that are more suited to a particular sport or area of performance as it was quite general. - More insight into the science behind flow training rather than just the basics. - The training could have been more engaging, possibly including more practical tests of flow? - Smaller group. - More information on psychology/brain function - I think the theory portion should be distributed as online learning materials beforehand so we can read it before. - Then Sunday would be more focused on a more practical approach and more engaging. I felt like there was a lot of theory on Sunday and a lot to absorb over a short time. Or perhaps more sessions. - More reading material to take home. - its good - Be more thought provoking - pitch/intro as part of an introduction email might give more time in the training for practical exercises |

**Supplementary Table 9.** Participants’ responses to training inclusions

| Question: What would you like to see in the training that wasn’t already in the workshop? |
| --- |
| - Not quite sure - I think it covered a lot. - Again - I wouldn’t add anything else it. From beginning to end, I was intrigued and will take a lot away from this and apply it to my practice - Perhaps a tip on staying in flow when you start in flow and then are distracted or managing expectations with the training. - Nothing really - How to challenge the lack of motivation or overthinking to get to flow - Further discussion about visualisation techniques for encouraging flow before you start your activity. A quick introduction into the purposes of different nervous systems before connecting them to how they assist flow would be interested to see how different senses can enhance or distract (or how they affect flow) - There’s nothing i really would like added - Case studies. Although time would be a factor - N/a - Exercises that we can apply in a daily basis - I would like to have experienced more of a discussion rather than a question-and-answer situation that seemed to be happening most of the time. It would be useful to talk specifically about the application of flow in my own practice. - Video testimonials and reverse engineering demonstrate flow - More practical application of the training and how we can change our personal circumstances to accommodate the training we received - More specific examples/stories from people who have used flow training. - :)) - The references - More practical exercises - comprehensive online learning materials beforehand. - Puzzles - It was awesome - its good - More hands-on activities - More exercises/ training sessions |

**Supplementary Table 10.** Participants’ perceptions of program effectiveness

| Item | N | Mean (SD) | Percentage of scores above 70% |
| --- | --- | --- | --- |
| The training reduced my stress in daily life beyond my activity | 26 | 4.31 (1.26) | 54% |
| The training helped me feel more confident in handling stressful situations? | 26 | 4.69 (1.32) | 57% |
| The training helped improve my performance | 26 | 5.00 (1.33) | 69% |
| The training helped me enjoy my activity more | 26 | 5.15 (1.35) | 81% |
| I feel that flow training can be applied to my activity | 26 | 5.77 (1.45) | 100% |
| The training helped me feel more confident in my performance | 26 | 5.27 (1.31) | 77% |
| Because of the training I feel more confident that I have the necessary skills to find flow in my activity | 26 | 5.46 (1.36) | 81% |
| Do you believe mental skills training can make a difference to your engagement? | 26 | 6.50 (0.81) | 100% |
| Do you believe this flow training will make a big difference? | 26 | 5.42 (1.17) | 81% |

Note: Items were scored 1-7, where higher scores denote more positive perceptions.

**Supplementary Table 11.** Participants’ responses to most effective aspects of the program

| Question: Please describe what you felt was most EFFECTIVE about the program? |
| --- |
| - Understanding how it works in the brain really helped me. as well as different contributors to the state of flow staring at a spot on the wall while widening your senses - That Cameron was able to give relevant examples and apply the concepts to each of our own chosen activities. Not everyone had sport as their activity and Cameron was able to help us understand how we can apply Flow to all aspects of our lives. - Increasing my motivation by reminding myself why i was doing it with a physical reminder - Identifying own motivations for activity - I like the changing the mindset (focusing less on the negative aspect on why you do something and finding the positive) - That it's something that can be incorporated into any activity in your life - ordered and methodical way of achieving flow. Practical application. - I learnt strategies that I can practice and implement in my own training. Since I am an anxious person, I found the section on challenge and motivation very helpful. - Wall of Confidence - Having different tools to pull from in order to have different ways of finding Flow. - Trusting your activity training + visualisation before performing, specifically visioning what you look like performing and how it feels to perform it successfully - Building up confidence and visualising the activity with enjoyment. - Learning about motivation - I found it very clear and engaging. I have used the motivation thing since the training, just in a small way to engage myself more in what I am doing - I'll make a mental note of my motivation for doing something and try and come up with some more intrinsic motivation. It is helpful to stay happier about doing some tasks that you might otherwise begrudge. - Practical exercises (e.g., balloons) as this demonstrated how focused and absorbed participants could become with a simple task - Information provided - the real-life examples explained - Find the balance between challenging myself and stressing myself - Step-by-step approach - Do not think at all and focus my attention on feel how every muscle was working - Preparation and focus - Know there is a flow which can help task be done more effectively. - How to focus and what to focus - Understanding the factors that influence the flow state and how to regulate them - Adding visual reminders for your best performance in the task from past and who you will be performing in the future |

**Supplementary Table 12.** Participants’ responses to most ineffective aspects of the program

| Question: Please describe what you felt was most INEFFECTIVE about the program? |
| --- |
| - I know courses are time poor, but i felt like the discussion times, should have had a "swap so the other person can talk" time, otherwise you get so involved you forget to swap talkers - The intro, but probably I had seen something similar on YouTube before, to get an idea of what it is about - Not sure. - I felt a bit confused in the application at first on how to do it - The visualisation exercise (wall) where the participant didn’t indicate any increase in confidence. (Bit of a flop, but handled well) - Sometimes it's difficult to change the mindset about being nervous, stress (i.e., over fears of injury) - It was too focused on sport as activity while it definitely covers much bigger areas in life than just that one field. - Nothing - Did not cater to my training specifically. As a dancer, the flow experience is very different to what it might be for other sports because there are so many corrections and feedback to remember when dancing. I would have liked if it was more catered towards the individual sport or if there was feedback on flow theory so that I could understand how it can work better for me. - None - Not having more of a scientific understanding of what exactly happens to the body in flow. And maybe not having someone to guide you through it - having to figure it out. - Writing down my motivations did not help me to find flow, as these motivations were already underlying in my performance pre flow training - Don't know - Not knowing what to do if the flow was inconsistent while engaging in the activity - I feel like it covered too much in too little time to be practical. I wonder whether it would be best presented over a series of workshops, where you can cover just one idea and practise that into your training and evaluate its effectiveness over a week before learning something new. I felt this training was very generic, and in order to use it for your specific task, and considering your own habits and psyche, more engagement was required on the part of the participant. However, it did give you a good idea of what flow is. - Still unsure how to get into flow in a sporting situation where you need to focus on what a competitor is doing and react/take proactive action. How does that active thought process does not pull you out of flow state - Time to practice - Hard to adapt its methodology into dance training - Not having enough content to take home to follow up with - Balloon activity - people misunderstood instructions - Thinking about time, control, performance - Nothing - How to have flow in daily work task not practical for me. Sometimes I can feel flow in simple tasks, or task I know well how to do. - n/a - None - n/a |

**Supplementary Table 13.** Participants’ responses to the benefits of the program

| Question: Please list the training benefits (i.e., how has it helped you? How has it made you feel?). |
| --- |
| - It has made me aware for the need to get out of my head and trust my natural motions more. - It mainly helped me to get more motivation for the activity itself (going out in shitty weather instead of finding excuses) - It has made me more confident in my performance ability. It has made me think about how I can apply flow and concentration skills to most aspects of my life. It has made me curious about my potential and I am excited to see how far I can go in my chosen activity and in others. - I feel more motivated when i use the technique of the physical reminder of why i am doing it - Find more flow. More enjoyment in task - I will become more aware of my mindset before and during the activity and to reframe my thoughts to be more constructive to getting into the flow. - More aware of my motivation in certain experiences more at ease. Confident. Utilising a good method to achieve more awareness and flow in everyday activities. - I have been able to put things in perspective more to achieve the optimum level of challenge. I have also enjoyed finding more intrinsic motivations. Both of these helps to reduce my stress and anxiety around the activity. - That there is such a thing as Flow and that I am not imagining it. - I have been able to perform without internal chatter in the brain, something I really struggle with. It has also made me feel more competent and less tired from sessions. - Trust in self and in my years of training. Able to recognise stress/tension in the body and feel motivated to let go of it. Ability to feel more confident in my future self after successfully executing difficult steps. - It has helped me directing my concentration & awareness back to the task. - Solidified my understanding of flow and how it works - I felt like it did help me get through a task (learning a new song) more effectively and with less struggle than previously. I didn't feel enlightened (it still felt like work) but I was engaged, and it did make the process easier, and I completed it quicker than before. I might have given up in the past whereas this time I broke up the learning into tiny tasks and it flowed a lot smoother. - More positive approach to training sessions, finding enjoyment, and seeking the state of effortlessness - Helped increase motivation - Becoming more conscious of my internal physiology - Motivation to try, confidence - Concentration in the task, understanding of my skills and capability, motivation to Create more flow moments - Relaxation and focus on everything other than the breath-hold - Keep Calm on task - It’s awesome - Learning to trust - Flow Training makes yo mindful of the flow state. Focus Improves. Visualize how you will do the new tricks before doing helps. - Coordination and picking queues improve. Re-focus when not being able to learn new tricks as you have been successful in the past in learning new tricks. positive reinforcement of thoughts |

**Supplementary Table 14.** Participants’ responses to whether the training helped increase awareness of flow or ability to find flow

| Question: Do you feel that since the training you are simply more aware of flow or do you feel that you can actual find flow more frequently or intensely? |
| --- |
| - More aware, but also more aware of how to find it - Main improvement so far: I now start applying it to different daily tasks - I feel at the moment I am simply more aware of Flow, but with time as I get better at flow, I will find it more frequently and intensely. - I feel more aware of it. I was playing videogames, and I realised after I was done that I had been in flow. - More aware and able to find flow. - Yes, I think I can recognize effective vs ineffective thoughts that will contribute to flow - I'm definitely more aware & will keep practising finding flow in my everyday tasks - A combination of awareness and then able to apply. - Yes, I am much more aware of flow and understand how I can practice the strategies so that I can find it more often - Yes - I am definitely more aware of flow, and I think I have been able to find it more effortlessly. - Both - I can recognise and appreciate the moments I find flow, but can also put myself into a headspace pre performance to prepare for flow - I can feel it's a work in progress. Initially I faced more challenges but could quickly realise they were caused by things I had written down as "adding unnecessary stress" during flow training. So, it helped me identify WHY I was more challenged - I am more aware, but found that I was already achieving a good sense of flow - I might have experienced flow more, but I'm still not really enjoying my activity. But I'm not hating it as much, so perhaps that's a win. - More aware and desire to find flow more frequently - more aware - More aware - Both - I feel that I can find flow, but I need to really focus all my attention in what I am doing in the precise moment - More aware but can prepare better which benefits 'finding' a flow state - Not sure - After the Flow training I learn to find flow on my daily task and at my workplace and its help me to be more productive - Yes, sometimes - Since the Training I'm more mindful of the flow and have learned to use it in favour. This applies to anything which we do in everyday life and not just the activity we chose. YES |

**Supplementary Table 15.** Participants’ perceptions of training

| Item | N | Mean (SD) | Percentage of scores above 70% |
| --- | --- | --- | --- |
| The training reduced my stress in daily life beyond my activity | 26 | 4.31 (1.26) | 54% |
| The training helped me feel more confident in handling stressful situations? | 26 | 4.69 (1.32) | 57% |
| The training helped improve my performance | 26 | 5.00 (1.33) | 69% |
| The training helped me enjoy my activity more | 26 | 5.15 (1.35) | 81% |
| I feel that flow training can be applied to my activity | 26 | 5.77 (1.45) | 100% |
| The training helped me feel more confident in my performance | 26 | 5.27 (1.31) | 77% |
| Because of the training I feel more confident that I have the necessary skills to find flow in my activity | 26 | 5.46 (1.36) | 81% |
| Do you believe mental skills training can make a difference to your engagement? | 26 | 6.50 (0.81) | 100% |
| Do you believe this flow training will make a big difference? | 26 | 5.42 (1.17) | 81% |

Note: Items were scored 1-7, where higher scores denote more positive perceptions.

**Table 16. Pearson Correlations between PFS scores and Aspects of Flow Measurement and Preliminary Outcomes**

| **Variable** | ***N*** | **Range** | ***M*** | ***SD*** | **Global Flow** |
| --- | --- | --- | --- | --- | --- |
| Flow Intensity | 52 | 1 to 7 | 4.90 | 1.33 | *r* = .699, *p* < .001 |
| Flow Duration | 52 | 1 to 100 | 60.23 | 24.80 | *r* = .311, *p = .*054 |
| Performance | 52 | 1 to 7 | 4.71 | 1.32 | *r* = .727, *p* < .001 |
| Choice | 52 | 1 to 7 | 5.39 | 1.20 | *r* = .534, *p* < .001 |
| Intrinsic Motivation | 52 | 2 to 7 | 5.19 | 1.22 | *r* = .892, *p* < .001 |
| Competence | 52 | 1 to 7 | 4.77 | 1.13 | *r* = .729, *p* < .001 |
| Stress | 52 | 1 to 10 | -3.32 | 1.29 | *r* = -.372, *p* < .01 |
| Well-being | 52 | 1 to 100 | 95 | 22 | *r* = .673, *p* < .001 |

*Note.* All values (except *p* values) rounded to 2 decimal points. Negative differences for stress represent positive reduction in scores.

**Table 17. Preliminary Assessment of Online Vs In-Person Flow Training**

|  | **Mean** | | **Percentage** | |
| --- | --- | --- | --- | --- |
| **Variable** | **Online** | **In-Person** | ***Online*** | **In-Person** |
| Flow (PFS) | 5.61 | 5.2 | +36% | +22% |
| Performance | 5.25 | 5.23 | +52% | +41% |
| Intrinsic Motivation | 4.38 | 4.90 | +8% | +5% |
| Well-Being | 107.00 | 100.91 | +57% | +12% |
| Ability to Handle Stress | 5.00 | 5.27 | +35% | +133% |
| Felt Stress | 2.00 | 2.14 | -18% | -39% |
| Felt Anxiety | 2.00 | 2.00 | -39% | -27% |

*Note.* All values (except *p* values) rounded to 2 decimal points. Negative differences for felt stress and felt anxiety represent positive reduction in scores.
