# Supplementary material for: Psychological Flow Training: Feasibility and Preliminary Efficacy of an Educational Intervention on Flow
Source: Int J Appl Posit Psychol. 2023 May 23:1–24. Online ahead of print. doi: 10.1007/s41042-023-00098-2 (PMC10204032; doi:10.1007/s41042-023-00098-2)
Supplement: Supplementary file 2 — (DOCX 19.3 KB) [file 41042_2023_98_MOESM2_ESM.docx]

**Method - Flow Experience Characteristics**

In addition to the PFS, participants were given direct questions (pre- and post-intervention) targeting flow experience characteristics (entry, duration, occurrence, intensity) that have been recommended for further examination (see Ellis et al., 2018; Engeser, 2012b; Jackman et al., 2017; Moneta, 2012; Swann et al., 2018). To assess *self-reported flow entry,* participants were asked: "The questions above are designed to measure ‘flow’ experiences. Flow is described as 'A total engagement in which nothing else matters, actions seem to flow effortlessly - simply participating in the act feels satisfying.' Based on your experience and responses to the above items, do you think that you experienced ‘flow’ in your recent task/activity?" The response was binary (i.e., '*yes*’, ‘*no*’, or ‘*unsure*') to ensure a discrete measure (see discussions around flow being a discrete and continuous construct; Norsworthy et al., 2021; Peifer & Engeser, 2021). If participants answered ‘*no*’ to self-reporting being in flow they skipped questions regarding frequency, intensity, and occurrence of flow. To assess self-reported *flow duration*, participants were asked: "What percentage of the time were you in flow in relation to the total time (length) of the activity?" A percentage score was designated for responses. To assess *flow occurrence* participants were asked: "Was the flow experience a single experience or did you or enter and exit it on multiple occasions within the activity?" Ordinal responses ('just once’, ‘on two or three occasions’, ‘multiple occasions') were provided. To assess self-reported *flow intensity,* participants were asked: "Referring back to the experience of flow, how strongly did you experience flow?"; a 7-point response scale was provided (ranging from *very weak* to *very strong*).

**Results - Flow Experience Characteristics**

***Flow Entry***

Participants self-reported '*yes*' (*n* = 8)*, 'unsure*’ (*n* = 8), and '*no*' (*n* = 8) during pre-intervention measures. In post-intervention measures, participants self-reported '*yes*' (*n* = 21)*,* '*unsure*' (*n* = 4), and *'no*' (*n* = 1)*,* suggesting that the perception of flow entry either increased (by 262%), participants were made more aware of flow states post-intervention, or both.

***Flow Duration***

On average, participants self-reported being in flow, within their given activity, 60.23% (*SD* = 24.8) of the time. Flow duration scores did not positively correlate with PFS scores (*r* = .311, *p = .*054).

***Flow Occurrence***

Participants self-reported entering and exiting flow *'once*' (*n* = 2)*,* on *'2 or 3 occasions*' (*n* = 8), and on '*multiple occurrences*' (*n* = 4) during pre-intervention measures. In post-intervention measures, participants self-reported being in flow '*once*' (*n* = 2)*,* on *'2 or 3 occasions*' (*n* = 13), and on '*multiple occurrences*' (*n* = 7), suggesting that entering flow 2 or 3 times was the most common occurrence of flow.

***Flow Intensity***

Participants self-reported a mean intensity score of flow of 4.9 (*SD* = 1.33), increasing by 11% post-intervention. Total flow intensity scores correlated with total PFS scores (*r* = .699, *p* <.001).  Also see S1, Table 16, for correlational statistics.

**Preliminary Assessment of Online Vs In-Person Flow Training**

Although examining the difference between online and in-person training was not a primary aim of the study—for the interested reader—we have included descriptive findings. Post-intervention, participants undergoing the training via Zoom (online) self-reported a mean *flow* score of 5.61 (+36%), *performance* score of 5.25 (+52%), *IM* score of 4.38 (+8%), *well-being* score of 107 (+57%), *ability to handle stress* score of 5 (+35%), *felt stress* score of 2 (-18%), and *felt anxiety* score of 2 (-39%); whilst in-person training self-reported a mean *flow* score of 5.2 (+22%), *performance* score of 5.23 (+41%), *IM* score of 4.9 (+5%), *well-being* score of 100.91 (+12%), *ability to handle stress* score of 5.27 (+133%), *felt stress* score of 2.14 (-39%), and *felt anxiety* score of 2 (-27%). See S1, Table 17. Participant data were too small to draw conclusions, though initial results suggest online training participants reported equally positive or improved results.
